# Effect of preoperative hair removal vs. no removal on surgical site infections: a systematic review and meta-analysis

**DOI:** 10.12688/f1000research.158369.1

**Published:** 2024-12-05

**Authors:** Abdulsalam Aleid, Saud Nayef Aldanyowi, Abdulmajeed Aljabr, Hasan Ali Abdullah Alaidarous, Zainab Aleid, Abdulaziz Alharthi, Mutlaq Alsubaie, Lama AlOraini, Abdulrahman Almoslem, Abbas Al Mutair

**Affiliations:** 1 King Faisal University, Al Ahsa, Eastern Province, Saudi Arabia; 2 King Saud bin Abdulaziz for Health Science, Riyadh, Riyadh, Saudi Arabia; 3 Albaha University, Albaha, Albaha, Saudi Arabia; 4 Alhada Armed Military Hospital, Taif, Makkah, Saudi Arabia; 5 National Guard Hospital, Al Ahsa, Eastern province, Saudi Arabia; 6 King Fahad Specialist Hospital, Buraidah, AlQassim, Saudi Arabia; 7 Almoosa Specialist Hospital, Al Ahsa, Eastern Province, Saudi Arabia

**Keywords:** Surgical site infection, hair removal, clipping, depilatory cream, razor shaving, meta-analysis.

## Abstract

**Background:**

The practice of preoperative hair removal has been debated regarding its role in Surgical Site Infection (SSI) prevention. This study aimed to compare the different hair removing modalities and investigate the effect of preoperative hair removal on SSI rates.

**Methods:**

A systematic review and meta-analysis were conducted according to PRISMA guidelines. Three databases—PubMed, Web of Science, and Cochrane Library—were searched for relevant studies comparing preoperative hair removal to no hair removal. Studies eligible for inclusion were randomized controlled trials (RCTs) and cohort studies reporting SSI rates. Odds ratios, mean differences, and p-values were analyzed using a random effect model.

**Results:**

Seventeen studies involving 5,407 patients were included. No statistically significant difference in SSI rates was found between the hair removal and no removal groups (OR = 1.066, 95% CI 0.646–1.758, p = 0.803). When comparing clipping to no hair removal, there was no significant difference (OR = 0.967, 95% CI 0.642–1.455, p = 0.870). Razor shaving was associated with higher skin damage and slightly increased SSI risk compared to clipping but not statistically significant (OR = 0.749, 95% CI 0.346–1.623, p = 0.464). Depilatory creams, however, were favored over razor shaving (OR = 3.235, 95% CI 1.543–6.785, p = 0.002), as they were linked to less skin damage and easier application.

**Conclusion:**

Preoperative hair removal does not significantly impact SSI rates. Clipping appears to be a safer alternative to shaving, while depilatory creams show promise as an effective, less damaging option.

## Introduction

Surgical site infections (SSIs) has been linked to influence patient morbidity, healthcare cost, length of hospital stays, and increased fatalities in extreme cases. ^1, 2^ The practice of removing axillary hair prior to surgery has been widely discussed and examined, particularly in relation to its impact on the rate of surgical site infections. ^3, 4^ Given the pressing need to evaluate the impact of preoperative hair removal techniques on surgical site infection outcomes, additional research has been recommended to definitively establish the effectiveness of these techniques. ^5, 6^ This systematic review and meta-analysis sought to compare the effectiveness of different pre-operative hair removing techniques in reducing the incidence of surgical site infections (SSI), with the goal of enhancing patient care and improving post-operative results.

## Methods

This systematic review and meta-analysis were carried out in strict accordance with the PRISMA 2020 guidelines (DOI: 10.6084/m9.figshare.27331266) as described by Page et al. (2021). ^7^ The study was also registered in PROSPERO (CRD42024547805).

### Study search

A comprehensive electronic search for medical literature began on February 26th, 2024, to investigate the effects of hair removal versus no removal on surgical site infection (SSI) rates. Predetermined keywords ensuring high-quality and relevant results were employed, including surgical site infections, hair removal, razor shaving, depilatory creams, and preoperative therapies.

The literature search was conducted across three databases: PubMed, Web of Science (WoS), and the Cochrane Library. Truncation symbols and relevant MeSH terms were incorporated to ensure the search was comprehensive. To ensure a comprehensive and unbiased literature search, two independent reviewers employed EndNote 20 to identify and exclude duplicates from the initial search results. Reviewers then screened the remaining articles’ titles and abstracts against predetermined eligibility criteria. Subsequently, full texts of relevant articles underwent scrutiny for content quality. Any discrepancies arising during the screening process were resolved through discussion or consultation with a third party.

### Study screening and selection

Methodological guidelines were followed during the study selection and data collection phases to guarantee the validity and dependability of the systematic review and meta-analysis as guided by (Kim, 2023), ^8^ and (Muka et al., 2019). ^9^ After conducting the literature search and screening process, studies meeting the predefined criteria were selected for inclusion.

Two independent authors assessed the full texts of the articles to determine eligibility for inclusion in the systematic review and meta-analysis. In cases of disagreement regarding the inclusion of studies, a third author was consulted to resolve conflicts and achieve consensus.

### Eligibility criteria

Inclusion criteria included studies comparing preoperative hair removal with no hair removal, hair removal using razor when compared to other modalities (e.g., clipping, depilatory creams), those examining outcomes related to surgical site infections (SSIs) or complications from hair removal. Study designs included in this meta-analysis were randomized controlled trials (RCTs) and cohort studies. The target population consisted of adult patients undergoing any type of surgery, with the primary outcome being SSIs and the intervention being hair removal versus no removal. Studies were excluded if they were not in English or lacked full-text access. Also, case reports, case series, commentaries, expert opinions, review articles, systematic reviews/meta-analysis, economic analysis, cadaver studies, narrative review, and any study with less than 10 patients were all excluded.

### Data extraction

After the review process, the eligible studies were subjected to data extraction using a standardized data extraction form that used study characteristics to analyze data from the articles. The form specifically highlighted the methods of research adopted by each study (study designs), the sample sizes used by each study, age, sex, the specific method used to remove hair, and the results obtained. The two reviewers worked independently to retrieve the data to avoid inaccuracies in the process.

### Outcomes measured

The primary outcomes focused on comparing different hair removal techniques individually against no hair removal, as well as assessing various combinations of methods, to determine which approach is most effective in preventing SSI.

### Statistical analysis

We conducted a meta-analysis using Review Manager version 5.4, pooling the dichotomous variables to calculate the odds ratio (OR). A p-value of 0.05 or lower was considered statistically significant, with 95% confidence intervals (CI). Heterogeneity was assessed using I ^2^, with significance determined by the p-value.

## Results

### Literature search

**Figure 1.**
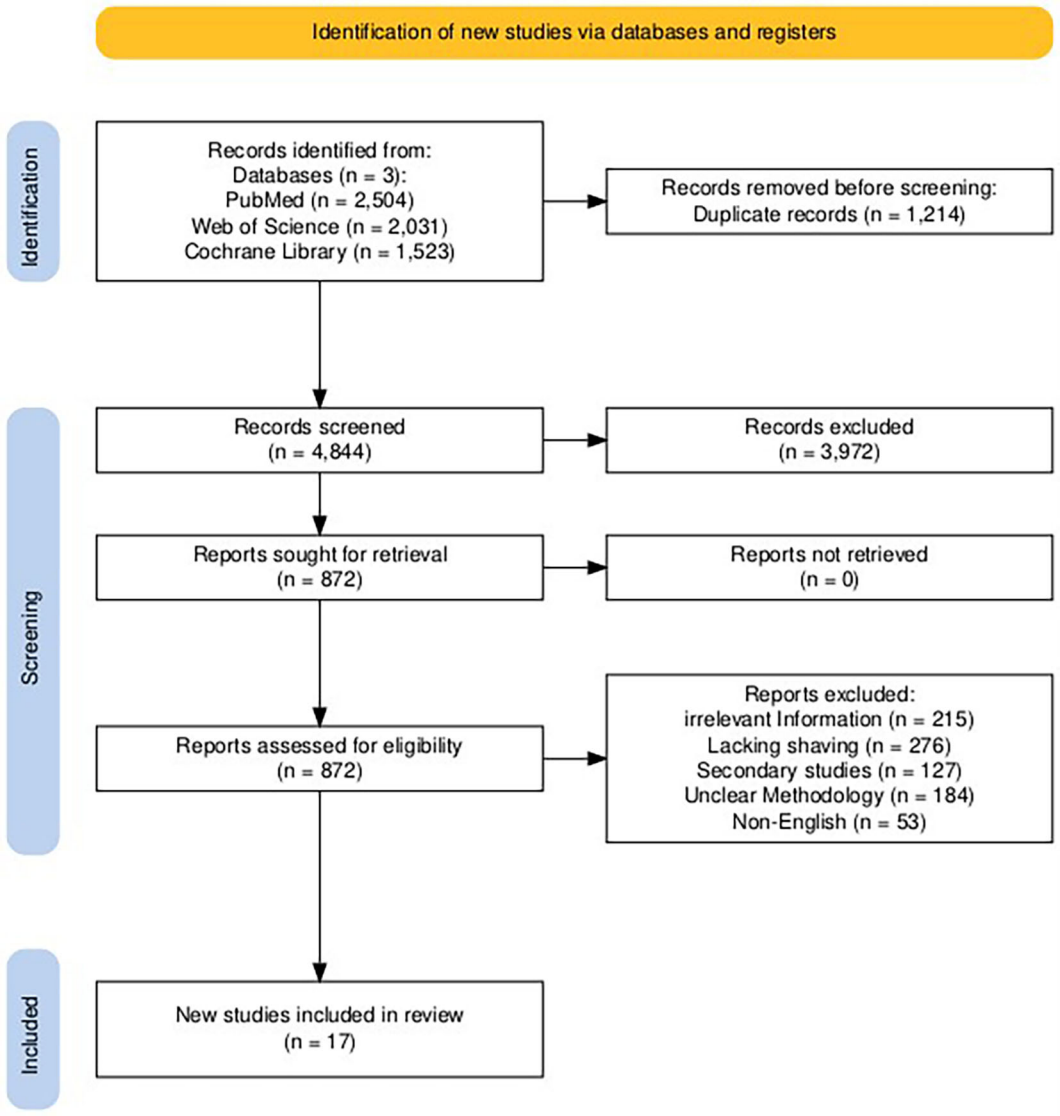
PRISMA flowchart of the literature search and studies selection process.

A comprehensive search across various databases identified 6,058 initial records. After removing duplicates (n = 1,214) and non-English publications, title and abstract screening excluded 3,972 articles for irrelevance. Full-text review of the remaining studies (n = 872) identified issues like irrelevant information, invalid methodologies, and insufficient data, leading to the exclusion of 855 articles. This rigorous process yielded 17 final studies that met the eligibility criteria and were included in this systematic review and meta-analysis ( Figure 1).

### Characteristics of the included studies

Seventeen studies met the eligibility criteria and were analysed for design, sample characteristics (sample size, age, gender), surgery type (e.g., abdominal, cranial), intervention methods (e.g., razor, clipping), follow-up period, and findings. The basic characteristics of the included studies are given in Table 1(extended data). Each study’s contribution to SSI prevention was assessed individually. Study designs included randomized controlled trials (RCTs), prospective RCTs, prospective comparative cohorts, prospective non-inferiority trials, and clinical trials. These studies encompassed 5,407 patients, with 265 developing SSI. Patient ages ranged from 6 months to 88 years, with a median of 44 years. The studies investigated various surgeries: cranial, spinal, abdominal, Lichtenstein hernia repair, intracranial, genital, appendectomy, chest, VP shunt insertion, and external/middle ear surgeries. Intervention methods included razor shaving, clipping, and depilatory creams. Follow-up periods varied greatly, ranging from 3 days to 10 months.

### Statistical analysis

**Figure 2.**
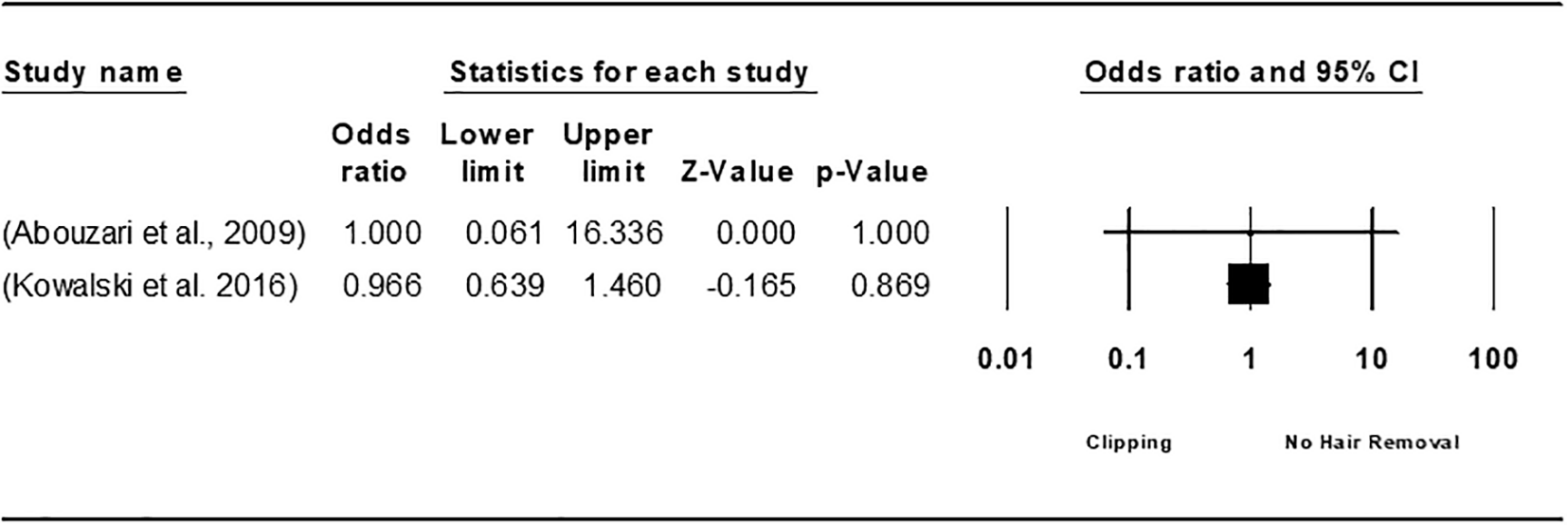
Forest plot of the effect of hair removal VS no removal on SSI.

**Figure 3.**
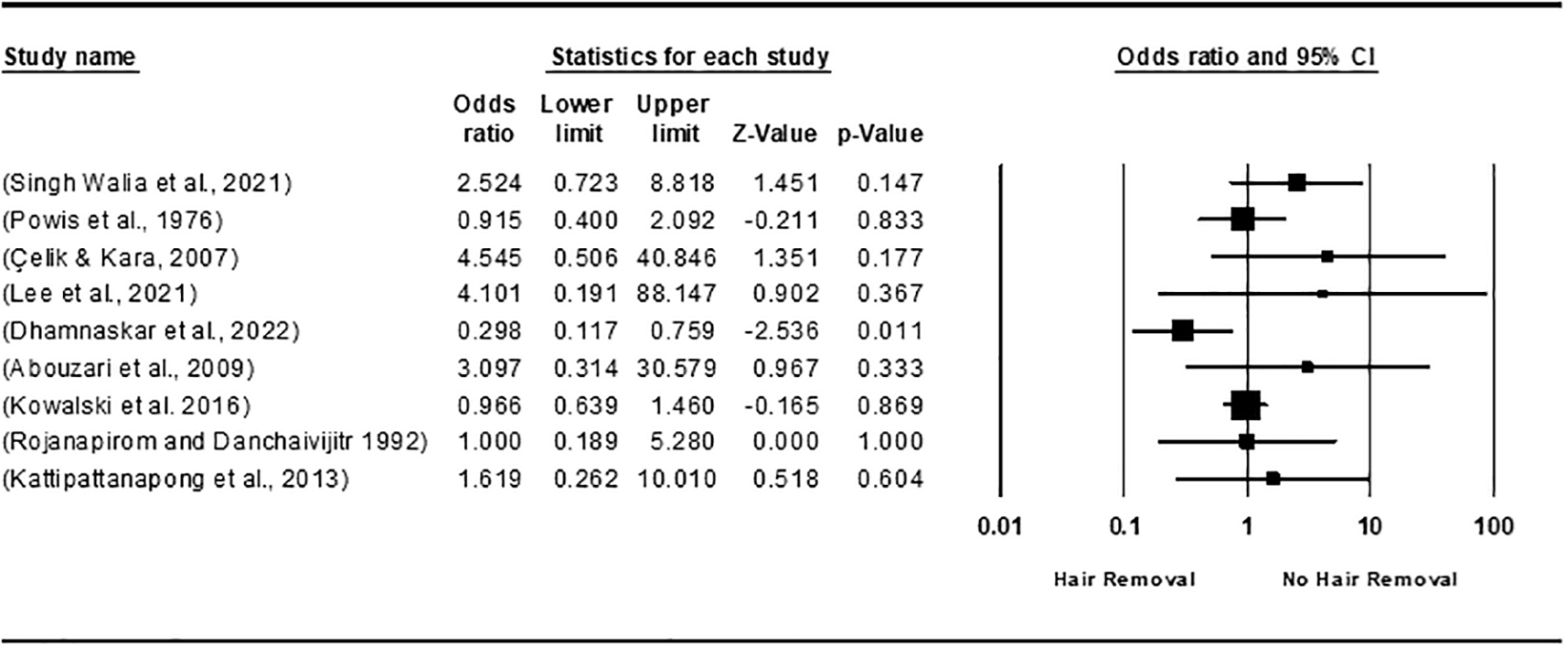
Forest plot of the effect of hair removal using clipping to no hair removal on SSI.

**Figure 4.**
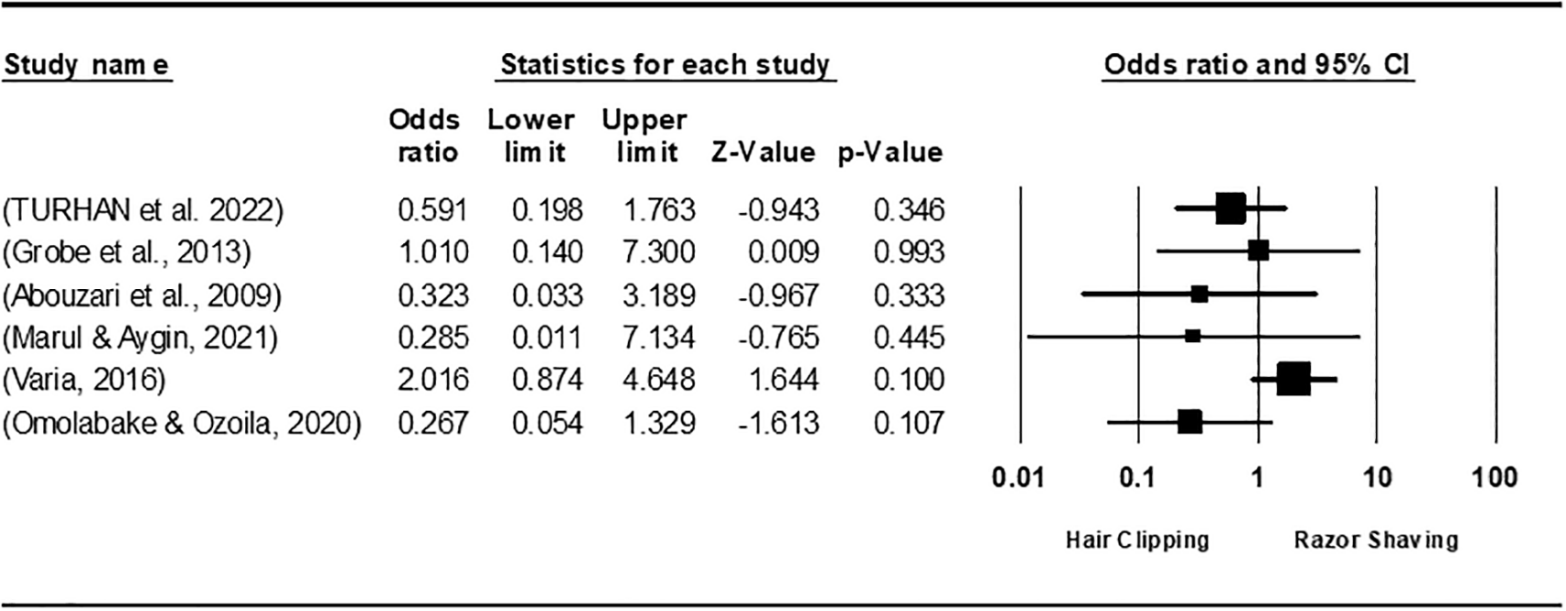
Forest plot of the effect of clipping and Razor shaving on SSI.

**Figure 5.**
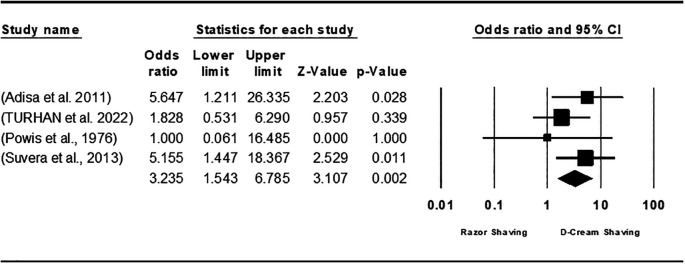
Forest plot of the effect of Razor shaving and using depilatory cream on SSI.

**Figure 6.**
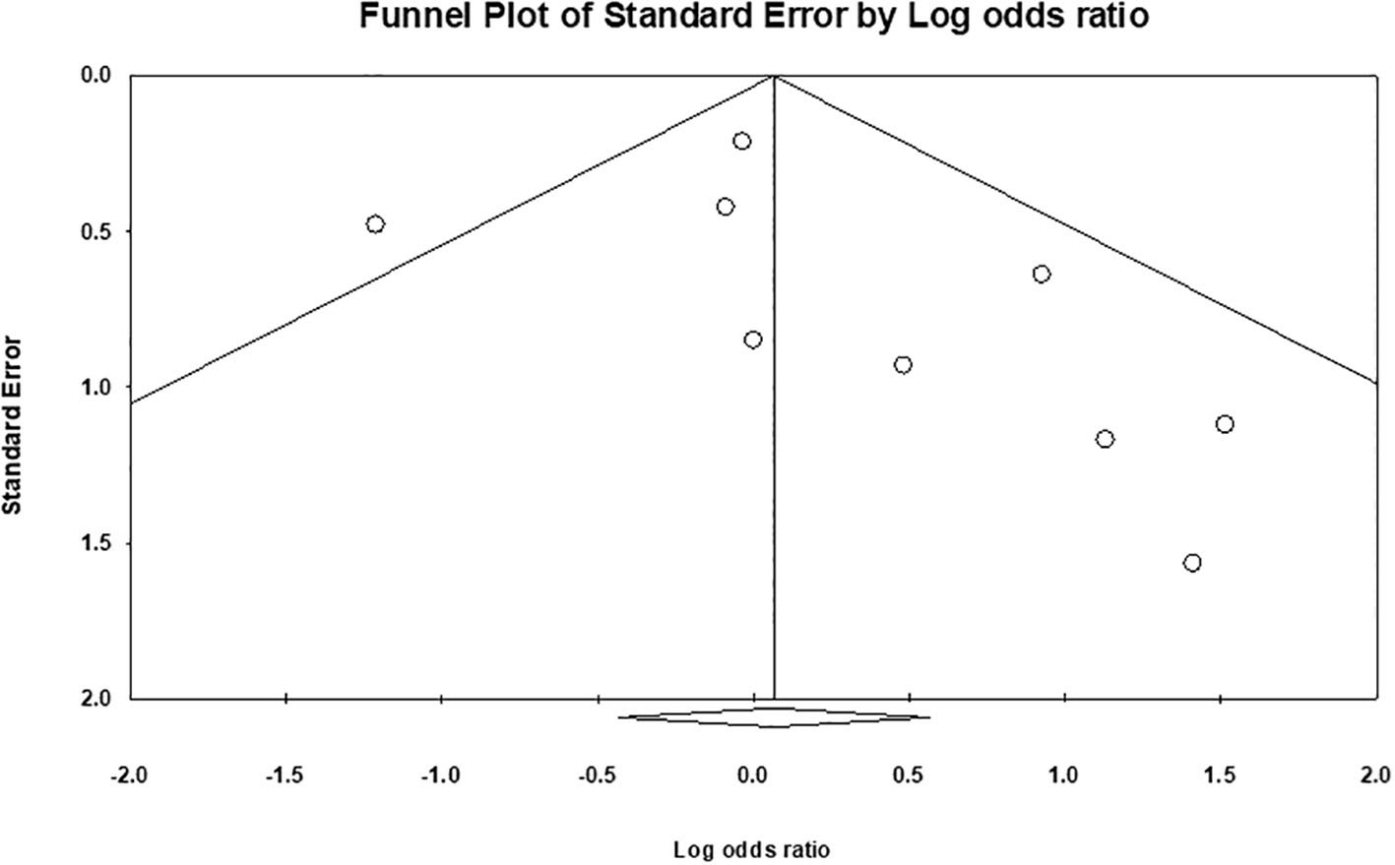
Publication bias for studies in hair removal vs no hair removal meta-analysis.

**Figure 7.**
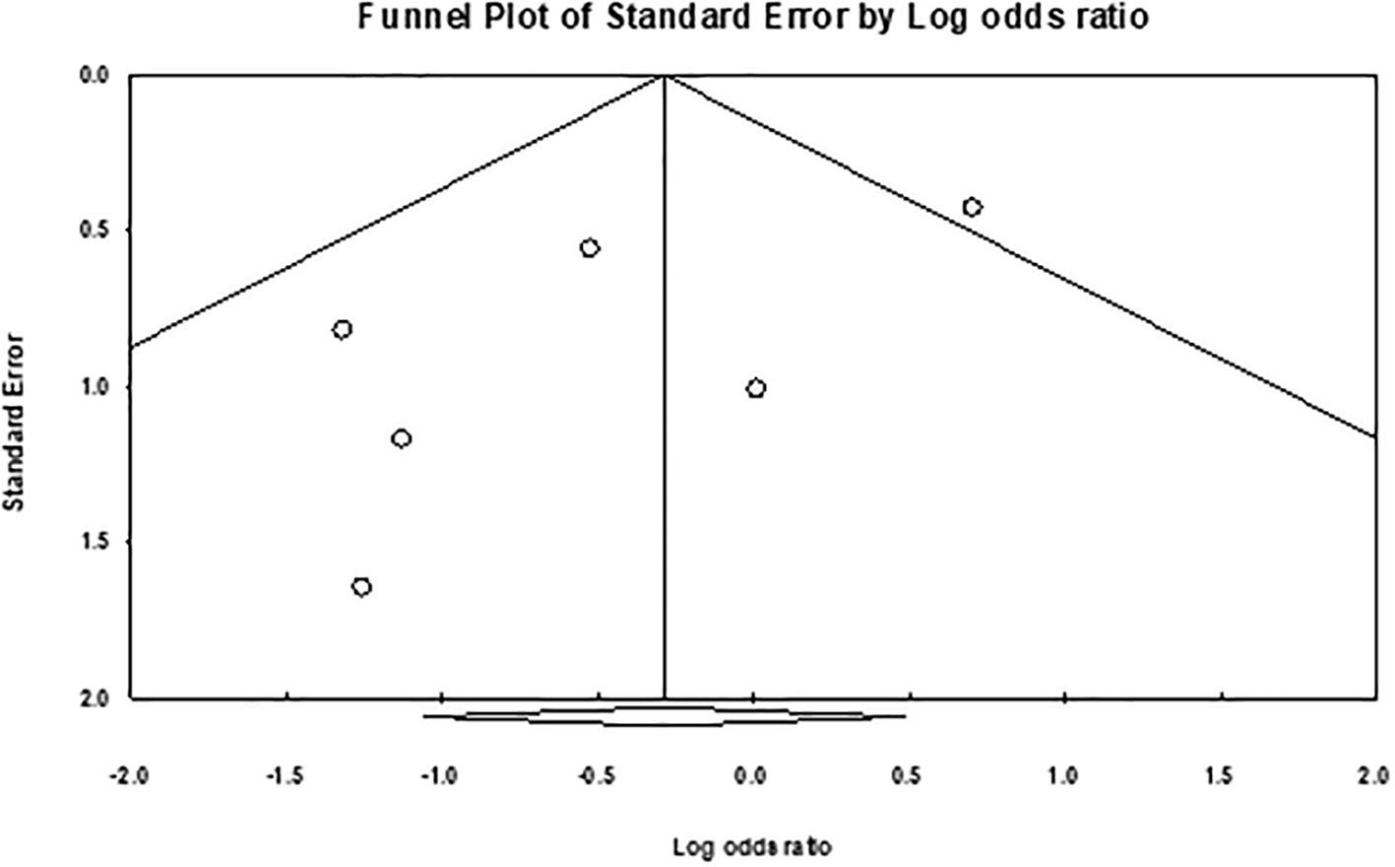
Publication bias for studies in clipping vs razor shaving meta-analysis.

**Figure 8.**
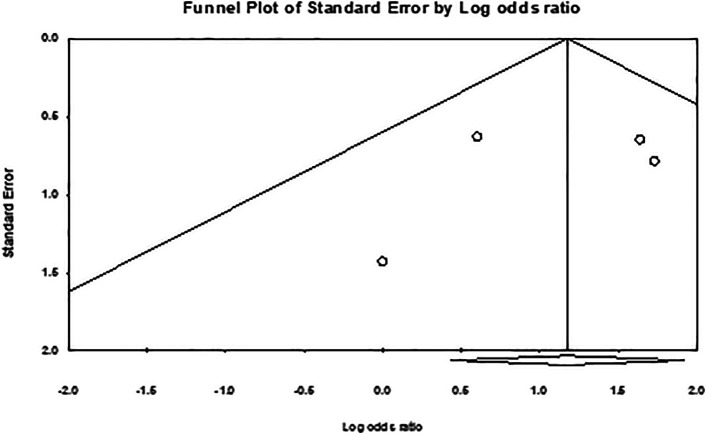
Publication bias for studies in razor shaving vs depilatory cream meta-analysis.

The analysis comparing hair removal versus no removal found no statistically significant difference, with an overall odds ratio (OR) of 1.066 [95% CI 0.646, 1.758; P = 0.803], and the heterogeneity was below 50% ( Figure 2). In a comparison of preoperative clipping to no hair removal, two studies, Abouzari et al. (2009) ^10^ and Kowalski et al. (2016), ^11^ showed no notable difference between the two methods, with an OR of 0.967 [95% CI: 0.642-1.455; P = 0.870] and no heterogeneity (I ^2^=0.0%; P= 0.981) ( Figure 3). Additionally, when comparing hair clipping to razor shaving, the analysis favored hair clipping but indicated no statistically significant difference, yielding an overall OR of 0.749 [95% CI 0.346, 1.623; P = 0.464], with heterogeneity remaining under 50% (I ^2^=34.363%; P= 0.179) ( Figure 4). In contrast, depilatory cream was preferred over razor shaving, resulting in an overall OR of 3.235 [95% CI; 1.543, 6.785; P = 0.002], and the heterogeneity among studies was zero (I ^2^=0.0%; P = 0.473) ( Figure 5). Overall, the meta-analysis exhibited low publication bias, with studies showing large effect sizes across analyses. The comparison of hair removal versus no hair removal did not demonstrate any defined publication bias, as the studies were moderately scattered with minimal concentration around the mean effect size in the funnel plots. Furthermore, subgroup analysis plots revealed a few large studies that were uniformly distributed from the mean effect size ( Figures 6, 7, and 8).

## Discussion

In recent years, numerous studies have explored the effect of preoperative hair removal on the occurrence of surgical site infections (SSIs). ^12– 15^ However, the results presented lack conclusiveness and consistency, leaving this intervention entirely obscure in the realm of surgical medicine. ^4, 16– 21^ Some studies have reported a general lack of significant difference between shaved and non-shaved groups. ^6, 14, 16, 22– 26^ Others have even reported possible risks of SSIs with razor-shaving. ^22, 27– 31^ Moreover, multiple studies recommended excluding hair removal from preoperative preparation practices unless necessary. ^6, 14, 20, 32^

Preoperative hair removal has long been considered a routine preventive treatment against surgical site infections. ^33^ However, more current studies have revealed a contrary opinion on this event. ^6, 15, 32, 34– 36^ The current review included studies comparing surgery with and without preoperative hair removal. The analysis found no significant difference in infection rates between the two groups. This suggests that removing hair before surgery may not offer any advantage in terms of preventing infections. Similarly, surgery with hair removal using clippers to surgery with no hair removal showed no significant difference in infection rates. These findings suggest that hair removal before surgery may be unnecessary. These findings support the results attained by Tang et al. (2001), ^37^ and Horgan et al. (1999). ^38^ Specifically, the study by Tang et al. (2001) ^37^ lacked a significant difference between the shaved group and the unshaved one in all parameters that were investigated using a sample size of 90 patients. Additionally, the study by Horgan et al. (1999) ^38^ presented no cases of SSI development in both the experimental and control groups.

Further analysis was done for the comparison groups involving different hair removal methods: shaving to clipping, shaving by using depilatory cream, and clipping by using depilatory cream. The primary outcomes were the development of SSI, whereas the secondary outcomes considered damage to the skin, effectiveness of hair removal, cost of the method, and simplicity of the procedure associated with each method in hair removal. In this meta-analysis, six studies were reviewed to compare hair clipping with razor shaving for preoperative hair removal. ^27, 39– 43^ The meta-analysis revealed no clear advantage between the two methods, though the results slightly favored clipping. Notably, in the razor shaving group, significant skin damage was observed, which increased the likelihood of surgical site infections (SSIs) due to the compromised integrity of the skin. A comparison of razor shaving and depilatory cream was conducted using data from four studies aimed at evaluating the two methods. ^40, 44– 46^ The findings revealed notable differences between them, with depilatory creams being generally preferred. Depilatory creams were found to offer greater ease of use, cause less skin trauma, and ensure effective hair removal. Due to these advantages, depilatory cream is recommended over razor shaving for preoperative hair removal, as it is linked to reduced skin damage and a lower risk of surgical site infections (SSIs). In a study conducted by Turhan et al. (2022), depilatory creams were compared to hair-clipping techniques. The researchers concluded that there was no significant difference between the two methods in terms of their effectiveness for hair removal. This similarity was observed in both the overall results and the effect size analysis. ^40^

The overall heterogeneity in these meta-analyses was relatively low, with moderate variation observed in the primary comparison of hair removal versus no hair removal and the analysis of shaving. This variation may stem from differences between the individual studies. For example, studies with comparable sample sizes, follow-up durations, and participant demographics, such as sex, tended to produce more consistent results. On the other hand, studies with greater differences in these factors showed higher levels of heterogeneity. ^47, 48^

The traditional belief that preoperative hair removal reduces infection has been challenged in recent years. ^6^ Contrary to this long-held view, recent studies, including findings from our meta-analysis, indicate that hair removal may not be essential, as there was no significant difference in infection rates between patients who underwent hair removal and those who did not. The study also suggests that if hair removal is needed, using clippers is better than shaving with a razor. ^6^ Razors may increase the risk of infection by causing nicks and irritation. Depilatory creams seem like a promising alternative because they remove hair without damaging the skin, but more studies are needed on their long-term effects. ^49^

Several limitations were observed in studies investigating the connection between preoperative hair removal and SSIs. One key issue was the inconsistency in the quality of the evidence, with some studies not utilizing proper blinding or control groups. Additionally, many studies were constrained by small sample sizes. These shortcomings highlight the need for more robust studies to better understand the effect of preoperative hair removal on SSIs. Future studies should focus on well-designed randomized controlled trials (RCTs) that implement standardized hair removal techniques and include larger patient populations to clarify its role in SSI prevention.

## Conclusion

This systematic review and meta-analysis revealed no substantial difference in surgical site infection (SSI) rates between patients who had preoperative hair removal and those who did not. However, razor shaving was linked to a higher risk of SSIs when compared to clipping, but it was not statistically significant. Clipping emerged as the preferred method for hair removal when necessary, while depilatory creams showed promise as a potential alternative to shaving. These results question the conventional view that preoperative hair removal prevents infections, suggesting it should be avoided unless deemed necessary.

## Ethical statement

Not applicable as this review involves already published studies and no ethical issue.

## Data Availability

No data are associated with this article. Repository name: Figshare, Effect of preoperative hair removal vs. no removal on surgical site infections: a systematic review and meta-analysis, DOI: https://doi.org/10.6084/m9.figshare.27331266.v2. ^50^ This project contains the following data:
-
Table 1: Basic characteristics of included studies Data are available under the terms of the Creative Commons Zero “No rights reserved” data waiver (CC0 1.0 Public domain dedication). Repository name: Figshare, Effect of preoperative hair removal vs. no removal on surgical site infections: a systematic review and meta-analysis, DOI: https://doi.org/10.6084/m9.figshare.27331266.v2. ^50^ This project contains the following data:
- PRISMA_hair removal_checklist Data are available under the terms of the Creative Commons Zero “No rights reserved” data waiver (CC0 1.0 Public domain dedication).

## References

[1] Hou Y Collinsworth A Hasa F : Incidence, and impact of surgical site infections on length of stay and cost of care for patients undergoing open procedures. *Surg. Open Sci.* 2023 Jan 1;11:1–8. 10.1016/j.sopen.2022.10.004 36425301 PMC9679670

[2] Costabella F Patel KB Adepoju AV : Healthcare cost and outcomes associated with surgical site infection and patient outcomes in low-and middle-income countries. *Cureus.* 2023 Jul;15(7). 10.7759/cureus.42493 PMC10455046 37637579

[3] Okoli CC Anyanwu SN Emegoakor CD : Does preoperative chemical depilation make any difference in postoperative wound infection? *Niger. J. Clin. Pract.* 2020 Sep 1;23(9):1318–1323. 10.4103/njcp.njcp_149_20 32913174

[4] Dohmen PM Konertz W : A review of current strategies to reduce intraoperative bacterial contamination of surgical wounds. *GMS Krankenhhyg. Interdiszip.* 2007;2(2). 20204082 PMC2831242

[5] Edmiston CE Jr Leaper DJ Barnes S : Revisiting perioperative hair removal practices. *AORN J.* 2019 May;109(5):583–596. 10.1002/aorn.12662 31025350

[6] Tanner J Melen K : Preoperative hair removal to reduce surgical site infection. *Cochrane Database Syst. Rev.* 2021;8(8):Cd004122. 10.1002/14651858.CD004122.pub5 34437723 PMC8406791

[7] Page MJ McKenzie JE Bossuyt PM : The PRISMA 2020 statement: an updated guideline for reporting systematic reviews. *BMJ.* 2021 Mar 29;372. 10.1136/bmj.n71 PMC8005924 33782057

[8] Kim G : How to perform and write a systematic review and meta-analysis. *Child Health Nurs. Res.* 2023 Jul;29(3):161–165. 10.4094/chnr.2023.29.3.161 37554084 PMC10415837

[9] Muka T Glisic M Milic J : A 24-step guide on how to design, conduct, and successfully publish a systematic review and meta-analysis in medical research. *Eur. J. Epidemiol.* 2020 Jan;35:49–60. 10.1007/s10654-019-00576-5 31720912

[10] Abouzari M Sodagari N Hasibi M : Re: Nonshaved cranial surgery in black Africans: a short-term prospective preliminary study (Adeleye and Olowookere, Surg Neurol 2008;69-72) Effect of hair on surgical wound infection after cranial surgery: a 3-armed randomized clinical trial. *Surg. Neurol.* 2009;71(2):261–262. 10.1016/j.surneu.2008.01.059 18440617

[11] Kowalski TJ Kothari SN Mathiason MA : Impact of hair removal on surgical site infection rates: a prospective randomized noninferiority trial. *J. Am. Coll. Surg.* 2016 Nov 1;223(5):704–11. 10.1016/j.jamcollsurg.2016.03.032 27687471

[12] Jacob KR Markose A Mathew A : A randomized controlled trial to compare the effects of two methods of preoperative hair removal on surgical site infections in patients undergoing elective tympanoplasty. *J. Evol. Med. Dent. Sci.* 2018 Sep 10;7(37):4095–4101. 10.14260/jemds/2018/916

[13] Waltz PK Zuckerbraun BS : Surgical site infections and associated operative characteristics. *Surg. Infect.* 2017 May 1;18(4):447–450. 10.1089/sur.2017.062 28448197

[14] Shi D Yao Y Yu W : Comparison of preoperative hair removal methods for the reduction of surgical site infections: a meta-analysis. *J. Clin. Nurs.* 2017 Oct;26(19-20):2907–2914. 10.1111/jocn.13661 27875033

[15] Lefebvre A Saliou P Lucet JC : Preoperative hair removal and surgical site infections: network meta-analysis of randomized controlled trials. *J. Hosp. Infect.* 2015 Oct 1;91(2):100–108. 10.1016/j.jhin.2015.06.020 26320612

[16] Dhamnaskar S Mandal S Koranne M : Preoperative surgical site hair removal for elective abdominal surgery: does it have an impact on surgical site infection? *Surg. J.* 2022 Jul;08(03):e179–e186. 10.1055/s-0042-1749425 PMC9345678 35928549

[17] Uçkay I Harbarth S Peter R : Preventing surgical site infections. *Expert Rev. Anti-Infect. Ther.* 2010 Jun 1;8(6):657–670. 10.1586/eri.10.41 20521894

[18] Hranjec T Swenson BR Sawyer RG : Surgical site infection prevention: how we do it. *Surg. Infect.* 2010 Jun 1;11(3):289–294. 10.1089/sur.2010.021 20518648 PMC4702440

[19] Hijas-Gómez AI Lucas WC Checa-García A : Surgical site infection incidence and risk factors in knee arthroplasty: A 9-year prospective cohort study at a university teaching hospital in Spain. *Am. J. Infect. Control.* 2018 Dec 1;46(12):1335–1340. 10.1016/j.ajic.2018.06.010 30025619

[20] Niel-Weise BS Wille JC Van Den Broek PJ : Hair removal policies in clean surgery: systematic review of randomized, controlled trials. *Infect. Control Hosp. Epidemiol.* 2005 Dec;26(12):923–928. 10.1086/505454 16417032

[21] Tucci G Romanini E Zanoli G : Prevention of surgical site infections in orthopaedic surgery: a synthesis of current recommendations. *Eur. Rev. Med. Pharmacol. Sci.* 2019 Apr 2;23. 10.26355/eurrev_201904_17497 30977890

[22] Kose G Tastan S Kutlay M : The effects of different types of hair shaving on the body image and surgical site infection in elective cranial surgery. *J. Clin. Nurs.* 2016 Jul;25(13-14):1876–1885. 10.1111/jocn.13149 26879246

[23] Rojanapirom S Danchaivijitr S : Pre-operative shaving and wound infection in appendectomy. *J. Med. Assoc. Thai.* 1992 Mar 1;75 Suppl 2:20–23. 1402495

[24] Lee DH Yoo S Shin E : Nonshaved ear surgery: effect of hair on surgical site infection of the middle ear/mastoid surgery and patients’ preference for the hair removal. *J. Audiol. Otol.* 2018 Jul;22(3):160–166. 10.7874/jao.2018.00101 29890817 PMC6103491

[25] Kattipattanapong W Isaradisaikul S Hanprasertpong C : Surgical site infections in ear surgery: hair removal effect; a preliminary, randomized trial study. *Otolaryngol. Head Neck Surg.* 2013 Mar;148(3):469–474. 10.1177/0194599812472297 23283828

[26] Lee YH Kwon YS Cho JM : Ventriculoperitoneal Shunt without Hair Shaving Using Absorbable Suture Materials. *J. Korean Neurosurg. Soc.* 2021;64(1):120–124. 10.3340/jkns.2020.0088 32492983 PMC7819786

[27] Varia DM Kacheriwala S : Pre-operative shaving versus trimming and their relation to post-operative surgical site infection (SSI), a randomized controlled trial. *Int. J. Res. Med.* 2016;5(3):151–154.

[28] Seidelman JL Mantyh CR Anderson DJ : Surgical site infection prevention: a review. *JAMA.* 2023 Jan 17;329(3):244–252. 10.1001/jama.2022.24075 36648463

[29] Love KL : Patient care interventions to reduce the risk of surgical site infections. *AORN J.* 2016 Dec 1;104(6):506–515. 10.1016/j.aorn.2016.10.002 27890056

[30] Çelik SE Kara A : Does shaving the incision site increase the infection rate after spinal surgery? *Spine (Phila Pa 1976).* 2007;32(15):1575–1577. 10.1097/BRS.0b013e318074c39f 17621202

[31] Walia DS Saini JR Mohi RS : Shaving before Surgery-Merits & Pitfalls. *Ann. Int. Med. Dent. Res.* 2021;7(1):24.

[32] Small SP : Preoperative hair removal: a case report with nursing implications. *J. Clin. Nurs.* 1996 Mar;5(2):79–84. 10.1111/j.1365-2702.1996.tb00231.x 8696601

[33] Marecek GS Weatherford BM Fuller EB : The effect of axillary hair on surgical antisepsis around the shoulder. *J. Shoulder Elb. Surg.* 2015 May 1;24(5):804–808. 10.1016/j.jse.2014.10.007 25487899

[34] Viera V : Preoperative skin antisepsis. *Medicina Intensiva. Med. Intensiva (Engl Ed).* 2018 Nov 5;43:18–22. 10.1016/j.medin.2018.07.019 30409678

[35] Richards A Zaben M Patel C : The need for hair removal in paediatric brain tumor surgery? *Br. J. Neurosurg.* 2024 Mar 3;38(2):346–348. 10.1080/02688697.2021.1872777 33455445

[36] Thapa N Basukala S Regmi SK : Postoperative surgical site infection after preoperative use of razor versus clipper for hair removal in inguinal hernia surgery: A quasi-randomized clinical trial. *Health Sci. Rep.* 2024 Jan;7(1):e1830. 10.1002/hsr2.1830 38274137 PMC10808942

[37] Tang K Yeh JS Sgouros S : The Influence of Hair Shave on the Infection Rate in Neurosurgery: A Prospective Study. *Pediatr. Neurosurg.* 2001 Jul 1;35(1):13–17. 10.1159/000050379 11490185

[38] Horgan MA Kernan JC Schwartz MS : Shaveless brain surgery: safe, well tolerated, and cost-effective. *Skull Base Surg.* 1999;9(04):253–258. 10.1055/s-2008-1058134 17171113 PMC1656773

[39] Adeleye AO Olowookere KG : Nonshaved cranial surgery in black Africans: a short-term prospective preliminary study. *Surg. Neurol.* 2008 Jan 1;69(1):69–72. 10.1016/j.surneu.2007.02.046 18054619

[40] Turhan VB Topcu R Yıldız A : Is there any difference between shaving versus clipping versus depilatory gel of hair removal for skin preparation before surgery in respect of wound infection? *J. Health Sci. Med.* 2022 Mar 3;5(2):564–567. 10.32322/jhsm.1025686

[41] Grober ED Domes T Fanipour M : Preoperative hair removal on the male genitalia: clippers vs. razors. *J. Sex. Med.* 2013 Feb;10(2):589–594. 10.1111/j.1743-6109.2012.02904.x 22908852

[42] Marul F Aygın D : Evaluation of the Effect of Two Different Hair Removal Methods Before Surgery on Surgical Site Infections. *Gazi Med. J.* 2021 Jul 1;32(3). 10.12996/gmj.2021.84

[43] Omolabake BI Ozoila KN : A comparison of postoperative wound infection rates after preoperative hair removal with razors versus clippers in a suburban setting. *Int. Surg. J.* 2020 Oct 23;7(11):3627–3632. 10.18203/2349-2902.isj20204662

[44] Adisa AO Lawal OO Adejuyigbe O : Evaluation of two methods of preoperative hair removal and their relationship to postoperative wound infection. *J. Infect. Dev. Ctries.* 2011 Oct 11;5(10):717–722. 10.3855/jidc.1527 21997940

[45] Powis SJ Waterworth TA Arkell DG : Preoperative skin preparation: clinical evaluation of depilatory cream. *Br. Med. J.* 1976 Nov 13;2(6045):1166–1168. 10.1136/bmj.2.6045.1166 791444 PMC1689580

[46] Suvera M Vyas P Patel M : Two methods of pre-operative hair removal and their effect on post-operative period. *Int.J. Med. Sci. Pub. Health.* 2013 Oct 1;2(4):885. 10.5455/ijmsph.2013.050720131

[47] Linden AH Hönekopp J : Heterogeneity of research results: A new perspective from which to assess and promote progress in psychological science. *Perspect. Psychol. Sci.* 2021 Mar;16(2):358–376. 10.1177/1745691620964193 33400613 PMC7961629

[48] Glasziou PP Sanders SL : Investigating causes of heterogeneity in systematic reviews. *Stat. Med.* 2002 Jun 15;21(11):1503–1511. 10.1002/sim.1183 12111916

[49] Anderson DJ Podgorny K Berríos-Torres SI : Strategies to prevent surgical site infections in acute care hospitals: 2014 update. *Infect. Control Hosp. Epidemiol.* 2014 Sep;35(S2):S66–S88. 10.1017/S0899823X00193869 25376070

[50] Aljabr A : Effect of preoperative hair removal vs. no removal on surgical site infections: a systematic review and meta-analysis. figshare.Figure.2024. 10.6084/m9.figshare.27331266.v2 PMC11729190 39810848

