# Supplementary material for: Effect of preoperative hair removal vs. no removal on surgical site infections: a systematic review and meta-analysis
Source: F1000Res. 2024 Dec 5;13:1487. [Version 1] doi: 10.12688/f1000research.158369.1 (PMC11729190; doi:10.12688/f1000research.158369.1)
Supplement: Peer review history

# Reviewer report: Reviewer response for version 1

**DOI:** 10.5256/f1000research.173945.r353578

**Published:** 2025-01-13

**Authors:** Francesk Mulita

**Affiliations:** 1 Department of Surgery, General University Hospital of Patras, Patras, Greece

I would like to congratulate the authors on their fascinating work regarding this interesting report entitled “Effect of preoperative hair removal vs. no removal on surgical site infections: a systematic review and meta-analysis”. The manuscript is well-written and the incorporated figures make the study easy to follow.

 I strongly recommend acceptance for indexing of the paper after minor revision.

 1) I would like a brief discussion on SSI as a postoperative complication after colorectal surgery

 2) The SSI is the most common postoperative complication after colorectal surgery, causing pain and suffering to patients. In addition, this complication has been associated with negative economic impact, increased morbidity, extended postoperative hospital stay, readmission, sepsis, and death. I would suggest adding this important information to the discussion section and consider citing the recently published articles

 Mulita F, et al., 2022 [Ref-1]

 Panos G, et al., 2021 [Ref-2]

 3) I want a brief discussion on complications after colorectal surgery, and the use of new enzymes such as Butyrylcholinesterase levels as a predictive marker for complications after this procedure.

 Consider citing the recently published article on a new biomarker (Buche):

 Verras GI, Mulita F, 2024 [Ref-3]

Are the rationale for, and objectives of, the Systematic Review clearly stated?

Yes

Is the statistical analysis and its interpretation appropriate?

Yes

If this is a Living Systematic Review, is the ‘living’ method appropriate and is the search schedule clearly defined and justified? (‘Living Systematic Review’ or a variation of this term should be included in the title.)

Yes

Are sufficient details of the methods and analysis provided to allow replication by others?

Yes

Are the conclusions drawn adequately supported by the results presented in the review?

Yes

Reviewer Expertise:

colon cancer, thyroid cancer, obesity, complications

I confirm that I have read this submission and believe that I have an appropriate level of expertise to confirm that it is of an acceptable scientific standard.

## References

[1] : Postoperative sepsis after colorectal surgery: a prospective single-center observational study and review of the literature. *Prz Gastroenterol* .2022;17(1) : 10.5114/pg.2021.106083 47-51 10.5114/pg.2021.106083 35371356 PMC8942007

[2] : Risk of surgical site infections after colorectal surgery and the most frequent pathogens isolated: a prospective single-centre observational study. *Med Glas (Zenica)* .2021;18(2) : 10.17392/1348-21 438-443 10.17392/1348-21 34080408

[3] : Butyrylcholinesterase levels correlate with surgical site infection risk and severity after colorectal surgery: a prospective single-center study. *Front Surg* .2024;11: 10.3389/fsurg.2024.1379410 1379410 10.3389/fsurg.2024.1379410 39229253 PMC11368738

# Reviewer report: Reviewer response for version 1

**DOI:** 10.5256/f1000research.173945.r346927

**Published:** 2025-01-10

**Authors:** Aygin Dilek

**Affiliations:** 1 Department of Surgical Nursing, Faculty of Health Sciences, Sakarya University, Sakarya, Turkey

This study aims to compare different hair removal methods and investigate the effect of preoperative hair removal on SSI rates. Keywords were determined according to PICOS and

 A systematic review and meta-analysis were conducted according to PRISMA guidelines. The number of databases was limited to three. Was grey literature reviewed or excluded? It should be stated.

 Randomized controlled trials (RCTs) and cohort studies reporting SSI rates were included. Rate ratios, mean differences and p values were analyzed using a random effects model. However, the quality scores of these articles are not clear. What was used as a quality assessment tool? A table showing the quality scores given to the articles should be added.

Are the rationale for, and objectives of, the Systematic Review clearly stated?

Yes

Is the statistical analysis and its interpretation appropriate?

Yes

If this is a Living Systematic Review, is the ‘living’ method appropriate and is the search schedule clearly defined and justified? (‘Living Systematic Review’ or a variation of this term should be included in the title.)

Yes

Are sufficient details of the methods and analysis provided to allow replication by others?

Yes

Are the conclusions drawn adequately supported by the results presented in the review?

Yes

Reviewer Expertise:

surgical nursing

I confirm that I have read this submission and believe that I have an appropriate level of expertise to confirm that it is of an acceptable scientific standard.
